# Notices, Reviews, &c.

**Published:** 1859-09

**Authors:** 


### NOTICES, REVIEWS, &c.

*Webster's Dictionary.*—The editor of Horne Tooke's Diversions of Purley, (London: Tegg & Co., 1857) in his additional notes to that work says that Dr. Webster's Dictionary is much superior to "every English Dictionary that has yet appeared; in which whilst abundance of valuable etymological information is supplied, fidelity and accuracy in recording the meanings according to actual usage is not sacrificed in order to accomodate them to a pre-conceived system or to etymological conjecture.

Scholars will be glad to learn that Messrs. George and Charles Merriam, of Springfield, Mass., have directed their efforts steadily towards making Webster's Unabridged Dictionary all that a scholar could desire it, and have just issued a new edition of Webster's Unabridged Dictionary, containing one thousand five hundred pictorial illustrations, beautifully executed. Nine thousand to ten thousand new words in the vocabulary. Table of synonyms, by Prof. Goodrich, in which more than two thousand words are carefully discriminated, forming a fuller work on English synonyms of itself, than any other issued, besides Crabb, and believed in advance of that, table giving pronunciation of names of eight thousand distinguished persons of modern times, peculiar use of words and terms in the Bible, with other new features, together with all the matter of previous editions. In one volume of one thousand seven hundred and fifty papes. Price, $6 50.

Specimen pages of illustrations and other new features will be sent on application to the publishers.

An English edition of Webster's dictionary appeared with the pictorial illustrations ten years since.

Prof. Goodrich first introduced the feature of synonyms in this country in connection with a popular dictionary, in Webster's octavo in 1847.

No other English dictionary ever contained or announced as to issue, a table giving pronunciation of names of persons, until after the above announcement.

Our readers will remember that all the words of disputed orthography in Webster's dictionary number only forty-two out of ninety thousand. Six millions of text books in our public schools, and a large proportion of the issues of the American press are according to Webster's spelling, and are daily and hourly familiarizing both the eye and the mind of the people to Webster's orthography, and there is but lit tle dount of its superseding all others.

"*Woman's home book of Health,*" by John S. Wilson, of Columbus Geogia, is a forthcoming work intended for mothers and families, with a chapter on the management of infants. If Dr. Wilson succeeds in teaching any considerable number of young mothers how to take a wise and humane care of the innocents committed to their charge, he will be one of the greatest benefactors of the age, for he will prevent a larger amount of human suffering and premature death, than can readily be computed.

*The Presbytery Reporter, and Presbyterian Expositor*, both of Chicago, *Godey's Lady Book* and Dixon's *Scalpel*, have failed to reach us.

*The Ladies Home Magazine*, of Philadelphia, *The Happy Home* of Boston, *The Home Monthly* and Mrs. Day's *Hesperian*, of San Francisco, an excellent nunmber for July are received.

